# Book Reviews

**Published:** 1803-06-01

**Authors:** 


### CRITICAL ANALYSIS

### OF THE

### RECENT PUBLICATIONS

### ON THE

### DIFFERENT BRANCHES OF PHYSIC, SURGERY, AND MEDICAL PHILOSOPHY.

---

*An Inquiry into some of the Effects of the Venereal Poison on the human Body, with an occasional Application of Physiology, Observations on some of the Opinions of Mr. John Hunter and Mr. B. Bell, and Practical Remarks; by* R. Sawrey, Surgeon, pp. 201. 8vo.

Many important points concerning the theory of the Lues Venerea still remain undecided; nor are these of slight importance, since, in several essential particulars, the practice must be directed by them. The Author of this Treatise professes it to be his intention to examine into these disputed points, to criticise freely the opinions of some of the most eminent writers and practitioners, and to endeavour to explain some of the difficulties which lie in the way of these inquiries.

As the points in dispute have often been controverted, we shall confine ourselves to a simple abstract of the pages before us.

In the Preface, the Author thus claims the liberty of free Inquiry, a liberty which, in medical subjects, is seldom disputed.— "In the execution of this work, which I have found to be difficult, the subjects of which I hope the Reader will think with me to be of some importance, I have been under the painful necessity of differing in opinion from two modern and eminent authors. But in the practice of medicine, it is above all things necessary that there should be no *dictator*. This science, so essentially connected with the happiness of man, for a long time was under the bondage of names, and only began to rise to its present improvements when its students claimed and exerted the independence of free inquiry."

Whether we have quite emancipated ourselves from this bondage of names may perhaps be questioned by those who have not yet been able precisely to understand the import of such terms as "specific action," "taking on a new action," "excitability," &c.

In the first chapter, the Author discusses the important question, Whether the same poison produces gonorrhœa and chancre? In this, as all our readers must know, the opinions of Mr. Benjamin Bell and Mr. John Hunter are at variance; the former gentleman considering them as different specific contagions, the latter as the same contagion, acting in a different manner according to the

 part part which it affects. First, with regard to Mr. Bell's opinion, the Author observes rightly enough, that the proper way of considering this question is to examine the matter of fact, whether gonorrhœa ever contaminates the system and produces lues venerea; for that chancre produces this effect is disputed by none, and, therefore, if gonorrhœa should be found to act in the same manner on the general habit as chancre, the identity of the poison in each case would be decided.

The question has often been agitated, and cases have been given directly in point. The Author gives his. The first is the case of a female infected with gonorrhœa unattended with chancre, by a person who was suffering under the same complaint. — When she was cured of this symptom, a venereal eruption and sore throat appeared, which yielded to mercury. The female *asserted*, that she had no connection with any one but the person from whom she received the gonorrhœa. The second case is very similar to the former. A person infected with gonorrhœa gave a female, whom he supposed he alone had injured, both gonorrhœa and chancre. On this case the Author makes the following singular remark, "he had not the smallest idea but that if she was injured, he was the cause; being perfectly of opinion that she had no intercourse with any other man. I am aware that such presumptions as these are very apt to be fallacious. But, however, there are cases in which, from peculiarity of circumstances, *the belief and conviction of a man of sound judgment, who is desired to be wary and discriminating on the subject, must have very great weight.*"

A medical attendant cannot, certainly, disclose every particular relating to such cases; but as the author does not pretend that these females had any claim to credibility on account of *character*, we conceive that a wary and discriminating examiner will not be disposed to lay much stress on evidence like this.

The third and fourth cases are more to the purpose. In each, a gentleman affected apparently only with gonorrhœa, and treated by injections, was attacked with decided symptoms of lues whilst under cure, and without receiving any fresh infection.

The author also appeals to the profession, whether similar cases do not sometimes occur, though rarely. On a subject of this importance our readers will allow us, we hope, to give the evidence of a practitioner of great experience, and a writer of celebrity.

"Some English writers have maintained that the virus which produces gonorrhœa is not the same with that which causes chancre or lues. They hold the opinion that the acrid virus which occasions gonorrhœa is generally different from the proper syphilitic poison, and therefore that the idea of a syphilitic blennorrhagy, (*virulent gonorrhœa*) is entirely void of foundation.

To this I answer, that though it is uncommon to see a clap produce syphilis, yet it is not so rare but that several examples of it may be found, especially in great towns. I have seen it in several instances, without being being able to detect any appearance of chancre chancre either in the thigh or on the genitals. These accidents happen principally after blennorrhagy with more violent symptoms than usual, and where a great extent of surface is affected. For this reason they are more frequent in women than in men. But of all the syphilitic blennorrhagies which I have ever seen accompanied with ulcers in the urethra, *every one of them was followed with symptoms of lues*, and of the most indisputable kind. The reason why blennorrhagy so seldom produces lues, is, that when the syphilitic virus is applied to the urethra, it excites only a superficial ulceration, and very rarely produces excoriation or ulcers, whence the poison may be absorbed into the mass of the blood. The mucous-membrane of this canal is defended by a large quantity of mucus, the secretion of which is further increased by the local irritation, and hence too the virus is diluted, the sides of the urethra are defended from its action, and ulceration is prevented. But if this secretion is suddenly stopped, either by the violence of the irritation or by improper injections, in nine cases out of ten, ulceration of the urethra will follow, and this will as certainly be followed by lues as syphilitic ulcers will be in any other part of the body.

If there was as copious a secretion of mucus between the prepuce and the glans as in the urethra, ulcers would be as rare in the one as in the other. In the cases in which the syphilitic virus occupies the corona glandis, and produces an unusual discharge of mucus, no ulcers are observed, but only a considerable tumour, accompanied with a copious discharge of puriform mucus like that which occurs in common blennorrhagy. Hence this complaint has been termed the *blennorrhagia glandis*." (*Swediaur Traité sur les Maladies Syphilitiques*, 1798, *tom. i.*

To return to the work before us. In the second chapter the Author explains the differences between the effects of gonorrhœa and chancre, principally from the difference in the structure of the parts, and the ready disposition in the arteries of the urethra to throw off any noxious virus by abundant secretion into the urethra and its lacunæ.

In the third chapter, Mr. Bell's arguments for the dissimilar nature of gonorrhœa and chancre are examined. Much of the proof must of course depend on the degree of credit to be given to the supposed evidence of facts brought forward; in other cases the facts may be admitted, but explained in a different manner. Mr. Bell lays much stress on the circumstance, that in many cases of inflammation occasioned by the sudden stoppage of a clap, the patients were cured without the use of mercury, and without the appearance of any syphilitic symptoms. Where this sudden check to the gonorrhœal discharge happens, it has been supposed, Mr. B. argues, that this "must necessarily throw the matter into the blood, and that pox must accordingly ensue from it. Were the matter of the two diseases the same, (he adds) this would happen in every instance; so that, when we can show that it seldom happens even in appearance, we are entitled, from this argument alone, to conclude that they are produced from two different kinds of contagion." The author answers this objection of Mr. Bell, and, so far, successfully, by shewing that a sudden check of the gonorrhœal discharge does *not* necessarily throw the virus into the blood. The product of gonorrhœa, the author remarks, " is formed by a disease in the vessels of the urethra, and is deposited from their mouths into the urethra and its lacunæ, from which it finds easy egress out of the orifice of that canal. There is no particular *reservoir* where a certain quantity of the poison is laid up at the beginning of the disease, which quantity is either to be discharged or conveyed into the system before a cure can be accomplished or the disease be removed from its seat. Be the discharge stopped sooner or later, quickly or slowly, by the interference of nature or art, it is not a necessary consequence that the quantity of this matter, which might otherwise have been discharged, is thrown into the circulating fluid, for, as formed, it is deposited into the urethra and its lacunæ, and finds easy egress out of the urethra."

The author continues his remarks on the much disputed case of *translation* of gonorrhœal virus from one part of the body to the other, and particularly to the eye, a supposed case of which is given by Mr. Bell. The author's chief objection to this is, that he cannot conceive how such a translation should have happened through the medium of the circulation, on account of the very long, roundabout course which any quantity of virus must pursue from the urethra to the eye, the particulars of which circuitous course he traces through the lymphatic, pulmonary, and aortal systems with as much accuracy as the ready candidate while under examination for his diploma. Now, without undertaking to defend Mr. Bell's individual case, it is certain that, however difficult it may be to conceive how sudden metastases from one to a distant part of the body should be performed through the involved medium of the circulation, it is full as difficult to account for it by any other hypothesis. Even in the simple cases of peculiar aliments affecting the excretions of the body, the course of (for example) that part of asparagus, which, when taken into the stomach, gives a peculiar odour to the urine in a few hours, is sufficiently circuitous, nor is it easy to understand exactly how this change takes place.

The title of the next chapter is, Practical Admonitions upon the Cure of Clap. The only practical admonitions that occur are, to use small quantities of mercury with a view of preventing clap from contaminating the blood, and to employ such injections as " from experience are known most often to relieve, without injuring the part."

The author now proceeds to the *philosophy* of the venereal disease or the truly theoretical part of the question, a subject which involves an infinite number of the higher and more difficult questions in physiology, and can only be fully elucidated when these mysteries of the animal economy are unveiled to us. On this account, and because it would would be doing injustice to the subject to attempt an abstract of it in the limits that we must allow ourselves, we shall pass over the remainder of this volume, in which, though ingenuity and acuteness are exhibited, the author is much more successful in refuting errors than in explaining difficulties; and the several objections against Mr. Hunter's opinions, which he brings forward, have been often urged and never repelled in a satisfactory manner.

---

*Attempt to investigate the Cause of the Egyptian Ophthalmia, with Observations on its Nature and different Modes of Cure; by* George Power, *Assistant Surgeon to the Twenty-third Regiment of Foot, or Royal Welch Fusileers.* pp. 72. 8vo.

The author speaks of this dreadful disease as an eye witness, having been attached to the medical staff of the army that served during the campaign in Egypt in the year 1801, and having had under his care several hundred patients afflicted with a variety of complaints incidental to the climate, as he informs us in the introduction to this treatise. Many opinions and conjectures have been formed as to the nature of the Egyptian ophthalmia; it has been described in some of the later French publications, and one or two pamphlets have been published in England on the subject, no single one of which, however, can be considered as sufficiently full and accurate.

The cause of this disease has been variously given by different writers; some have attributed it to the mechanical effect of the burning sands of the desert blown against the eye, already strained by the vivid reflection of light and heat from the ground; others, to a nitrous or saline quality in the air; others, to sudden alternations of heat and cold, or rather to the excessive chill dews in the evening succeeding to the parched burning atmosphere of the day; and some have distinguished varieties of the disease, as produced in one case by a combination of all the above circumstances of local irritation, with those of general debility; and in another case, as evidently symptomatic of excessive bilious acrimony in the primæ viæ. This last opinion is advanced with great force in a small but excellent paper on this subject, published by Cit. Bruant, and inserted in *Desgenettes Histoire Médicale de l'Armée de l'Orient.*

Mr. Power begins by noticing several of the supposed causes of this Ophthalmia, many of which he entirely rejects.

The sands he thinks by no means an adequate cause of the complaint, for the following very cogent reasons: First, because the Bedouin Arabs are exempt from it, and even the Europeans of general Baird's army, on marching across the Isthmus of Suez, did not suffer from it; secondly, because the disease was chiefly contracted in the night, when the heavy dews prevented the dust from floating; and, thirdly, because the disease has occurred in places where neither of these causes could have operated.

It has long been remarked that the cultivators of rice in Egypt were particularly subject to blindness; but here too, as has often been observed, the exhalations from the inundated fields in which this this grain is cultivated, are much more likely to produce the disease than any fancied qualities of the grain itself.

The author then gives his own hypothesis. "As the immense quantities of animal and vegetable substances which abound in Egypt," he observes, "when acted on by great heat and moisture, cannot fail to pass into putrefactive fermentation, putrid effluvia must there assume the highest degree of malignity, the deleterious effects of which are obvious. . . . . . . . It fortunately, however, happens for the inhabitants of Egypt, that these effluvia become susceptible of new changes while buoyant in the atmosphere by spontaneous decomposition, whereby their component parts, becoming disengaged, are either reduced to their first principles, or by taking a new arrangement, form compounds by combination with other substances in the atmosphere. Thus the earthy and saline substances that abound in the Egyptian atmosphere are produced, whilst the swarms of insects that are propagated, probably tend to correct the putrescence of the air by a passive as well as an active agency, which it is not necessary here to insist upon."

For the sake of his chemical readers, it would have been desirable however if the author had insisted somewhat upon the active and passive agency of swarms of insects in correcting the putrescence of the air, as it is an hypothesis as curious as novel. Still however we do not come to the point, why Egypt should be peculiarly infested with ophthalmia, for the above remarks apply equally well to Surinam or Batavia. The author continues. "The ammoniacal and fixed alkaline salts, either in a nascent state or combined with different acids whilst floating in the wind or deposited with the dews, may tend to occasion an ulceration of the fauces, together with the peeling of the skin from the face and hands, and from their pungency must be peculiarly destructive to the eyes."

What the *nascent state of a fixed alkali* is, we yet have to learn; and even with regard to a supposed *saline* state of the Egyptian atmosphere, before we admit the fact, we must enquire what are the experiments on which this assertion is advanced? For, in the present state of chemical science, we look for something more than the old hypotheses concerning the *nitre of the atmosphere*.

"The argillaceous and calcareous earths, which abound in the atmosphere either in a separate state or combined with sulphuric or carbonic acid, and which were supposed by the French surgeons to be peculiarly destructive to the eyes, may also be *thus* accounted for."—How accounted for? Are these earths also in a *nascent state*, and generated by the mass of vegetable and animal putrefying matter?

But we suppose he means, that when certain winds, or perhaps any violent winds prevail, part of the dry materials of the soil are converted into dust, and this dust of course may consist of sand, of nitre, if the soil contains this salt, and of any other casual ingredient: it is of some importance, however, to distinguish between clouds of dust, and the regular constitution of the atmosphere in which which that dust is floating. With regard to the peculiarly injurious effect of the calcareous and argillaceous part of Egyptian dust, we must do the author the justice to say, that it is an opinion of Cit. Savaresi, and is supported by this writer on the following very singular proof: "L'expérience* prouve que ces deux substances & leurs bases occasionnent sûrement l'ophtalmie. En effet je les ai introduites apres les avoir pulvérisées, dans les yeux de divers chiens, qui devinrent presque aveugles le lendemain de l'operation. . . . . . . J'ai vu deux grenadiers se jetter, en plaisantant, de la chaux qui porta sur leur visage & entra dans leurs yeux; ils eurent une ophtalmie qui les obligea de venir à l'hôpital.—Experiment shews that these two substances, (clay and chalk) as well as their bases, will infallibly produce ophthalmia. I have actually introduced them, previously rubbed to powder, into the eyes of several dogs who became almost blind the day after the operation. . . . . . I have seen two grenadiers throw lime at each other, in jest, part of which struck the face and entered the eyes: the consequence was an ophthalmia, which obliged them to come to the hospital." With due respect to Cit. Savaresi, it hardly seems a very fair experiment to almost blind a number of poor dogs by stuffing chalk or clay into their eyes, and to infer from it that the particles of these earths, floating in the air, will have the same effect; and unless he can prove that *quick lime* is also floating in this heterogeneous Egyptian atmosphere, the innocent amusement of these two grenadiers will prove nothing more than what every common bricklayer's labourer has often experienced to his cost, namely, that it is bad for the eyes to get lime into them.

The next section of the pamphlet before us deserves attention. The author is still aware that all the predisposing or exciting causes of ophthalmia which he has enumerated, do not apply very exclusively to Egypt, and the question to be determined (for the fact is undoubted) is, the reason why the Egyptians are peculiarly obnoxious to this disease?

The answer to this he finds, partly in the corporeal and mental debility induced by the heat of the climate, partly by the abuse of the tepid and cold bath, excessive venery, opium, tobacco, and poor diet: But the following appear the causes peculiarly Egyptian: "The extensive steril plain that is constantly presented to the eye, bounded only by the horizon: its glowing surface strongly reflecting the rays of the sun, which torture the eye by impressing too great a quantity of light on the retina; at the same time that the acting organ finding nothing to relieve the view, or to afford an idea of distance, becomes unavoidably excited beyond its proper sphere of action." This is highly probable and sufficiently a local cause. The last clause, however, though ingenious, may perhaps be doubted; that is, whether it has a tendency to debilitate the eyes, to be in

* Memoires sur l'Egypte, Seconde Partie, p. 347.

in the habit of directing the sight to very distant objects. We remember that Vaillant asserts, that from being naturally short and weak sighted, he acquired length of sight and strong power of vision by travelling in the boundless and sultry plains of Caffraria; and certainly the strength of a seaman's eye is proverbial.

The next local cause of ophthalmia in Egypt is, "the custom of sleeping at night in the open air, imbibing with every inspiration, and absorbing at every pore, the putrid virus contained in the descending dews. What the peculiar nature of this putrid virus may be, I shall not take upon me positively to decide. But when the similarity of circumstances in which the various diseases of Egypt are found to arise, as well as the different and even opposite circumstances in which they show themselves are considered, it will appear highly probable that a putrid virus is the chief efficient and common cause of disease. . . . ."

"Thus, in a system peculiarly debilitated, and unable to resist all its powers combined, it produces that highly putrid fever called Plague. In a patient less relaxed, as the habit of body determines the disease either to the surface of the skin or to the intestines, an Eruptive Fever or Dysentery is produced; and when the putrid virus is but partially applied, to the eyes for instance, or to the mouth, or even on the surface of the body, Ophthalmia, ulcerated fauces, or ichorous blotches on the skin ensue."

The Author here opens a very interesting field for speculation, by this opinion on the common origin of the Plague, Dysentery, and Ophthalmia. The idea is ingenious at least, and merits attention. Ought it not to happen, however, on this hypothesis, that the symptoms of these three diseases should be mutually convertible, according to the force of the exciting cause? and should not ophthalmia be as common a symptom of plague as bubo or carbuncle?

In the next section the Author gives the evidences of the contagious nature of the Ophthalmia. They are very few in number, but very forcible. The fact of its occurring at Gibraltar, mentioned in Mr. Edmonstone's pamphlet, (which we noticed in a former Number) is very striking in support of the opinion that this disease is contagious.

The symptoms are then described. The Author thinks that the disease may be divided into the acute and chronic, with several inferior varieties. The attack begins with a slight itching in the eye, to which succeeds increased secretion of tears, followed by a dimness of sight, owing to the deposition of a mucilaginous matter on the cornea. Often the disease proceeds no further if due attention is paid to the patient, if his constitution is good, and he can be indulged with rest, and can avoid the light. If, however, the complaint continues, the secretion from the surface of the eye acquires great acrimony and produces excessive pain; then follows inflammation of the conjunctiva with protrusion of the eye-ball, which renders it very difficult to close the eye-lids, whilst the impression impression of light becomes excessively painful. If the disorder is not checked, the whole forehead suffers most acute pain, increased at every pulsation of the carotids, and the patient feels apprehensive that the very sutures of his skull will separate.

Tumours are formed in the parotid glands, which sometimes degenerate into foul abscesses, and every symptom appears of high inflammatory fever. At last suppuration within the ball of the eye follows, the whole of this organ becomes an abscess, the cornea bursts, and the eye is destroyed for ever; or else the purulent matter being confined, affects the general health; diarrhœa or dysentery supervene, and the case terminates at last fatally in the highest state of hectic debility.

A very troublesome and distressing symptom, even in the favourable cases, generally occurs; it is a very large œdematous swelling of the eye-lids, enlarging them so much as to obstruct vision very materially. Specks and opake knots on the cornea also are frequent on the subsiding of the inflammation.

The Egyptian doctors use a variety of remedies for this complaint, many of them whimsical and superstitious. They are very fond of scarifying the eye-lids, which, as it is used indiscriminately, appears to produce nearly as much harm as good. Much advantage is derived by a cold poultice of the leaves of the kali plant simply beat up with a little cold water.

The plan of cure adopted in the British hospitals was generally the antiphlogistic, both general and topical, but varied according to circumstances. In short, it appears that the treatment was much the same as it would have been in any other case of ophthalmia, some regard being had to the situation of men exposed to fatigue, and other debilitating causes. Blisters applied for a few hours as rubefacients, the author speaks of in high terms of commendation, especially in that state of great local debility and spasmodic contractions of the eye-lids and adjacent muscles, which was often left in delicate habits long after the inflammatory symptoms had been removed.

Even when the globe of the eye contained an evident quantity of pus and the cornea seemed ready to burst with the tension, if the patient could be placed in circumstances favourable to health, and his constitution was good, he sometimes recovered by the use of topical discutients, and even the sight was completely restored.

The Author then mentions a remedy which he was induced to use in his own case, having had too much reason to lament the frequent failure of the other plans of treatment. It is the use of opium internally, which seems by his account to have produced the most signal advantage. Having prevailed upon Mr. Davis (of the 26th Dragoons) to use this remedy himself, which was followed with a speedy cure, the author adds: "It is needless to say how eager we felt to communicate to our patients the advantages we had derived from this excellent medicine. The opium was administered according to the plan of cure hereafter laid down," (a quarter of a grain grain every four or six hours) "and it is a fact no less surprizing than true, that in the space of a month from the adoption of this remedy we were enabled to restore to the army almost every ophthalmic patient in a state of convalescence, or of perfect health."

The Author laments in the Introduction to this interesting little work the loss of his port-folio, which contained all his cases, and we think that his readers have equal cause of regret.

As the Author has more than once mentioned Cit. Bruant's paper, we shall extract what this physician says concerning the plan of cure, as most of our readers may not have an opportunity of seeing the original.

"This disease is frequently cured by the simple operation of Nature, and without any assistance from art; and indeed we may affirm with truth, that nothing so much opposes the cure as too great a profusion of remedies, especially topical. Some patients have been relieved by an eruption coming on at the temples; others, and the greater number, by a slight diarrhœa; and hence, to act conformably to the views of Nature, I have encouraged a discharge from the bowels during the whole duration of the disease, by employing tamarinds or other laxative tisanes.

The treatment has been varied, according to the different species of ophthalmia before described. When the affection is local, and the inflammation is the leading symptom, we employ cold lotions and revulsives of every kind. With this view great advantage should be derived from general bleeding, but the decided bilious temperament of our patients has prevented us from using this remedy, and besides it is strongly counter-indicated in soldiers weakened by the hardships of a protracted war.

"We have not been able to use local bleedings hitherto," *(it does not appear why)* "though they might be safer and not less advantageous than the other; at least we have the example of the inhabitants of the country, who use it with success in the outer angle of the eye. . . . . We have hitherto contented ourselves with removing from the eye every irritating cause, and above all, light. When the pain is severe we apply some emollient remedy, but sparingly, because the relaxation which it produces makes the tumefaction very obstinate, and much retards the cure. Besides, a blister to the nape of the neck is of more service in this case, especially when the pain is not confined to the eye, but affects the whole head. When the inflammation abates, we begin to use resolvent collyria, the strength of which we gradually increase, and with which we compleat the cure.

"When it is discovered that the ophthalmia depends on foulness of stomach, evacuants must be had recourse to. . . . . . In the third species of ophthalmia which I have described," (where the inflammation is very slight, and there is no swelling, but the complaint consists of several nervous symptoms, spasm of the eye-lid, of the globe of the eye, and excessive susceptibility to light) "besides using tonics internally, I employ external antispasmodics, none none of which answer so well as blisters; whereas, in the purely inflammatory ophthalmia, their use is very indirect. They are best applied behind the ears, and this also is the place selected by the Egyptians for the cautery, when they use fire in inveterate disorders of the eyes." *Histoire Médicale del Armée de l'Orient, par Desgenettes.*

